# Optimization of the size and location of the FOVs for CBCT capture of impacted lower third molars

**DOI:** 10.1186/s13005-025-00518-5

**Published:** 2025-06-07

**Authors:** Marc Anton Fuessinger, Maximilian Frederik Russe, Leonard Simon Brandenburg, Marc Christian Metzger, Johannes Schulze, Stefan Schlager, Jonas Wuester, Wiebke Semper-Hogg

**Affiliations:** 1 Department of Oral and Maxillofacial Surgery, Albert Ludwig University Freiburg, Hugstetterstr. 55, D-79106 Freiburg, Germany; 2 Department of Radiology, Albert Ludwig University Freiburg, Hugstetterstr. 55, D-79106 Freiburg, Germany; 3 Department of Oral and Maxillofacial Surgery, Albert Einstein University Ulm, Albert-Einstein-Allee 11, Ulm, Germany

**Keywords:** Cone beam CT (CBCT), Field of view (FoV), Imaging

## Abstract

**Background:**

Cone beam computed tomography (CBCT) is an established diagnostic tool for impacted wisdom teeth (third molars (3 M)) in proximity of the mandibular nerve canal. This study aims to define the minimum field-of-view (FOV) size and its localization to reduce radiation exposure. As reference, the chin rest of the CBCT device was used.

**Methods:**

Three-dimensional CBCT data sets were used to analyze the bilateral positions and dimensions of the wisdom teeth. A total of 215 wisdom teeth from a study population with a mean age of 21 years, including data from 82 male and 58 female patients, were mapped. By transformation into a common coordinate space using the device’s chin rest as a joint denominator, the optimal size and location for uni- and bilateral capture of the wisdom teeth were determined, for both best-case and worst-case scenarios with regard to patient positioning.

**Results:**

The minimal FOVs for the lower 3 M capture were H 23.5 mm × R 35.4 mm in the best-case scenario assuming optimal patient positioning and H 35.4 mm × R 36.6 mm in the worst-case scenario with rotational deviation along the transversal axis. For the upper 3 M, the minimal FOVs were H 29.9 mm × R 29.2 mm in the best-case scenario and H 38.6 mm × R 35.6 mm in the worst-case scenario. Unilateral capture of both the upper and lower 3 M required FOV dimensions of H 51.7 mm × R 39.8 mm and H 44.8 mm × R 36.8 mm, respectively. For bilateral capture of all four 3 M, the best-case FOV was H 44.8 mm × R 84.8 mm and the worst-case FOV was H 51.7 mm × R 85.6 mm.

**Discussion:**

This research provides indication-specific FOVs for uni- and bilateral imaging of the upper and lower 3 M. Taking into account optimal clinical practices for CBCT imaging, this study aims to propose clinically feasible FOV dimensions while meeting the technical specifications of commonly used CBCT devices. Clinical application of the results may help reduce radiation exposure of patients receiving CBCT imaging of the wisdom teeth. Transfer of the present results to other CBCT devices requires further research.

**Trial registration:**

The study is registered in the German Trial Register with the number DRKS00026149, 2024/02/21.

## Introduction

Cone beam computed tomography (CBCT) is an established tool in the diagnostics of pathologies of the head and neck, especially within the fields of dentistry and maxillofacial surgery [1, 2]. Due to the lower economical and spatial requirements of CBCT systems in comparison to conventional computed tomography (CT), which is usually only available in hospitals and dedicated radiological offices, CBCT has been widely adopted in dental offices [3, 4]. The operation of CBCT systems requires less resources and training. The diagnostic value for imaging hard tissue structures is comparable to conventional CT while, depending on the diagnostic protocols used, exposing the patient to lower amounts of ionizing radiation [4]. The radiation exposure is significantly influenced by the region of interest (ROI) and the applied field of view (FOV) [5]. The size of the FOV is an independent factor in radiation exposure during CBCT [6–8]. Similar observations can be made for different ROIs due to tissue-specific effects on the radiation dose [9–11].

Retention of the third molars (3 M) occurs in up to 80% of young adults [10]. Their removal is one of the most commonly performed outpatient surgical procedures [12]. Panoramic radiography (dental panoramic tomography, DPT) is the recommended basic imaging tool prior to surgical tooth removal. Preoperative CBCT imaging of the 3 M is essential after identifying potential risk factors, such as proximity to critical anatomical structures (e.g., inferior alveolar nerve), the complexity of the tooth’s root morphology, and the likelihood of impaction, all of which may not be adequately assessed through 2D imaging [10, 12–16]. Recent studies have shown an increased sensitivity and specificity of CBCT compared to panoramic radiography in diagnosing external root resorptions of second molars (2 M) due to impacted 3M [17–19].

Current CBCT devices include standard programs (e.g. for the molar regions), but there are no FOVs tailored in both position and size to specific anatomical regions. Pakbaznejad Esmaeili et al. have demonstrated the possible optimization of the FOV for imaging impacted maxillary canines [20]. Ilo et al. recently suggested a specific FOV for the lower 3 M based on anatomical landmarks [21].

The aim of our retrospective study was to determine if it is possible to define optimal cylindrical FOVs for CBCT imaging of single 3 M, pairs of 3 M (on the same side or in the maxilla/the mandible), or all four 3 M by using a large set of pre-existing CBCT images.

## Methods

### Patient selection

Using the order entry database for the radiology department of the University of Freiburg’s Center for Dental Medicine, a set of patients was selected that had received CBCT imaging of the jaw and face as part of clinical routine and with appropriate indication. Images were captured using the J Morita 3D Accuitomo F170. CBCT scans were acquired during routine clinical practice with varying clinical justifications and fields of view. Despite these variations, all scans were reconstructed with a standardized voxel size of 250 μm to minimize potential effects on dimensional measurements within the region of interest.

**Fig. 1:**
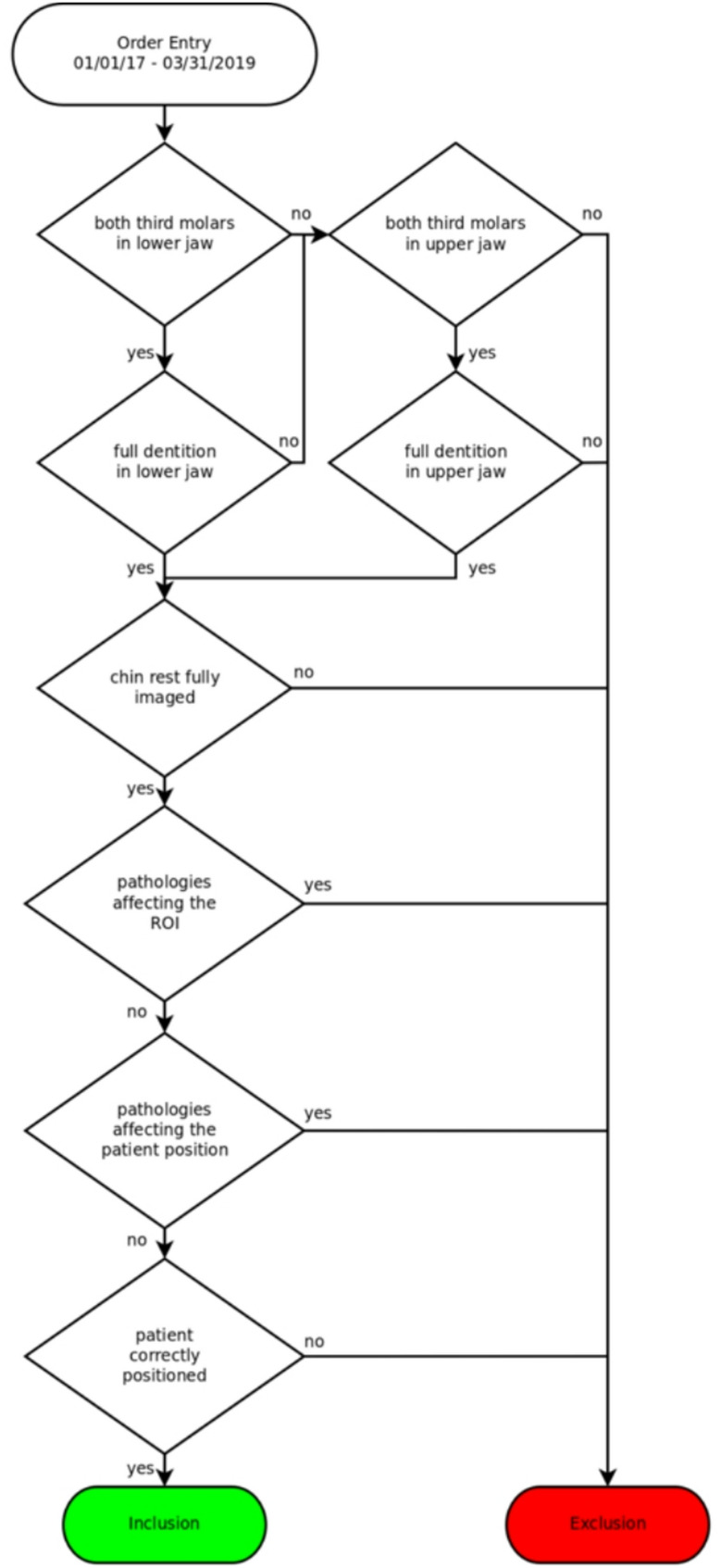
Selection process

The CBCT images of these patients were screened using the department’s Picture Archiving and Communication System (PACS). Patients were included if their CBCT scans showed at least two 3 M in one jaw or all four 3 M in both jaws. Patients were excluded if at least one 3 M was not imaged completely. Patients had to have a complete dentition in the examined jaw and no pediatric patients were included in the study. Datasets that included tumors, fractures, or other distorting pathologies of the jaw that might influence the anatomical position of the 3 M region were also excluded. Furthermore, patients were excluded if they showed any pathology or deformity of the imaged skeleton or surrounding soft tissue that would affect the patient’s position in the CBCT device or if the device’s chin rest was not completely included in the image. The selection process is depicted in Fig. 1.

The study was authorized by the ethics committee of the Albert Ludwig University Freiburg under the identification number 21-1188 defining that no participation consent was required.

### Data acquisition

**Fig. 2:**
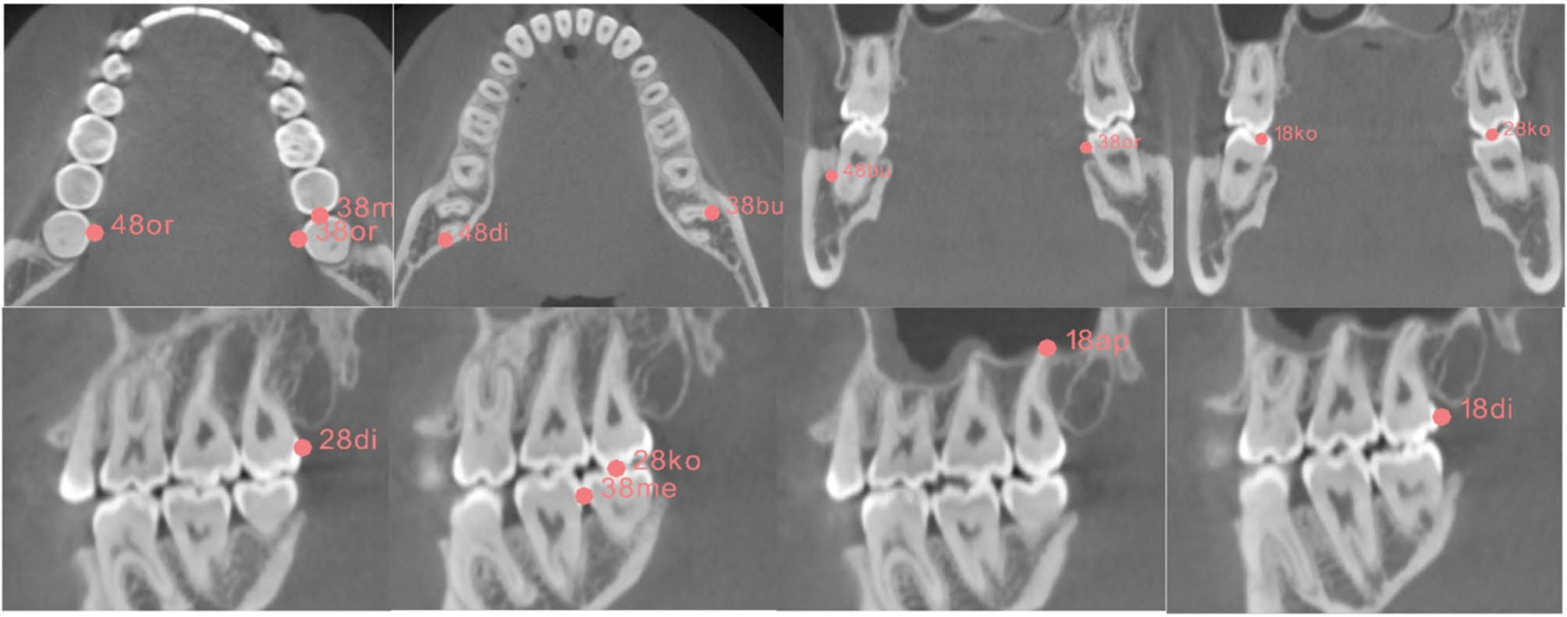
Mapping of the outer boundaries of the 3 M

The images were exported into DICOM format and later converted into NIFTI format facilitating collaboration and data analysis. The anonymization of the images was carried out by the hospital’s internal PACS system as part of the export function. For every 3 M, the dimension and location of the tooth were mapped by setting marker points in the most apical, coronal, distal, mesial, and buccal and oral extremities, as depicted in Fig. 2, by using the software 3D-Slicer [22]. Additionally, the position of the CBCT device’s chin rest as a universal point in relation to the device was marked. A total of 14 landmarks (12 anatomical landmarks parametrizing the molar ROI and 2 landmarks denoting the corners of the chin rest) was placed by two raters (resident and senior physician) throughout the sample. To account for asymmetries, the data was augmented with the mirrored counterparts, mirrored on the midsagittal plane of the data coordinate system.

### Data alignment

A single data set was chosen as a reference set and optimally aligned along the Frankfurt plane, the midsagittal plane, and the base of the mandible. All further transformations were based on this reference set.

In the study, two methods of spatial standardization were used to position patients, with the goal of evaluating both best-case and worst-case scenarios for variability in patient positioning.

#### Best-case scenario

This scenario is achieved by applying both translation and rotation around the corners of the chin rest. By incorporating rotation, the positioning accounts for more precise alignment of the patient’s head relative to the chin rest, which minimizes variability and allows for a more accurate representation of the patient’s anatomy in the standardized coordinate system.

#### Worst-case scenario

In contrast, the worst-case scenario involves only translating all configurations to the center of the chin rest corners without any rotation. This results in a less precise alignment, leading to greater variability since the original data is simply standardized by the common coordinate system without considering the optimal orientation of the patient.

In summary, the best-case scenario utilizes both translation and rotation to achieve optimal patient positioning, resulting in minimized variability, while the worst-case scenario relies solely on translation, leading to greater variability in the data.

### Translation and rotation

**Fig. 3:**
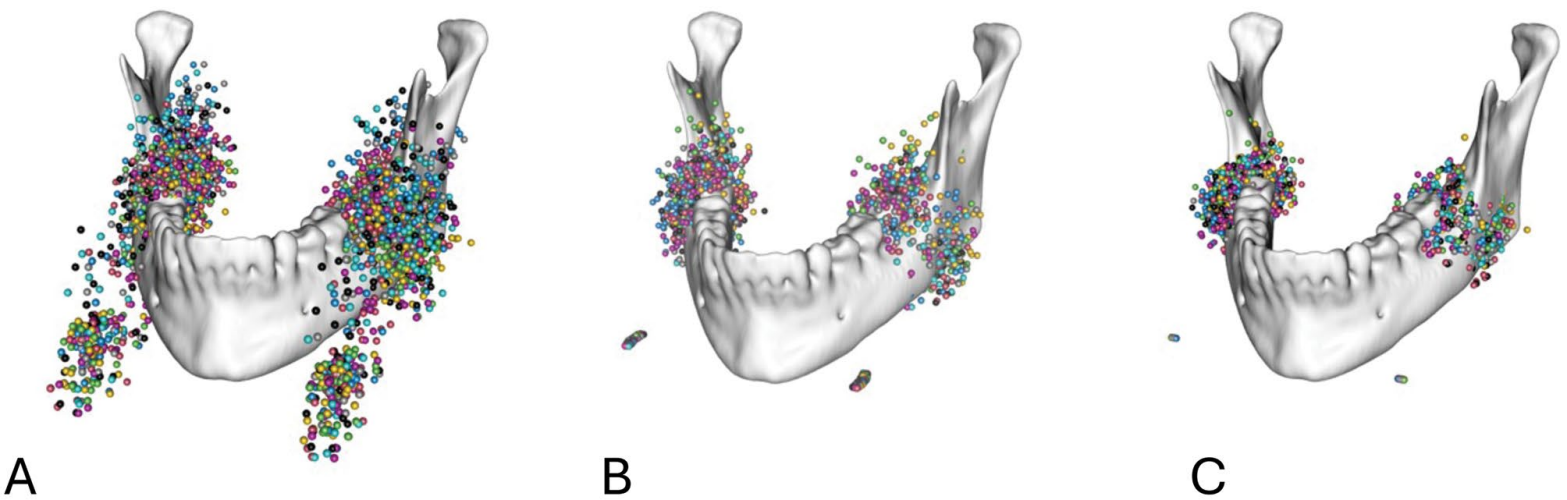
Markups for the 3 M of the mandible in relation to the mandible (**A**) before spatial alignment, (**B**) after spatial alignment by translation to a common coordinate system, and (**C**) after spatial alignment by translation to a common coordinate system and rotation around a transversal axis defined by the chin rest

**Fig. 4:**
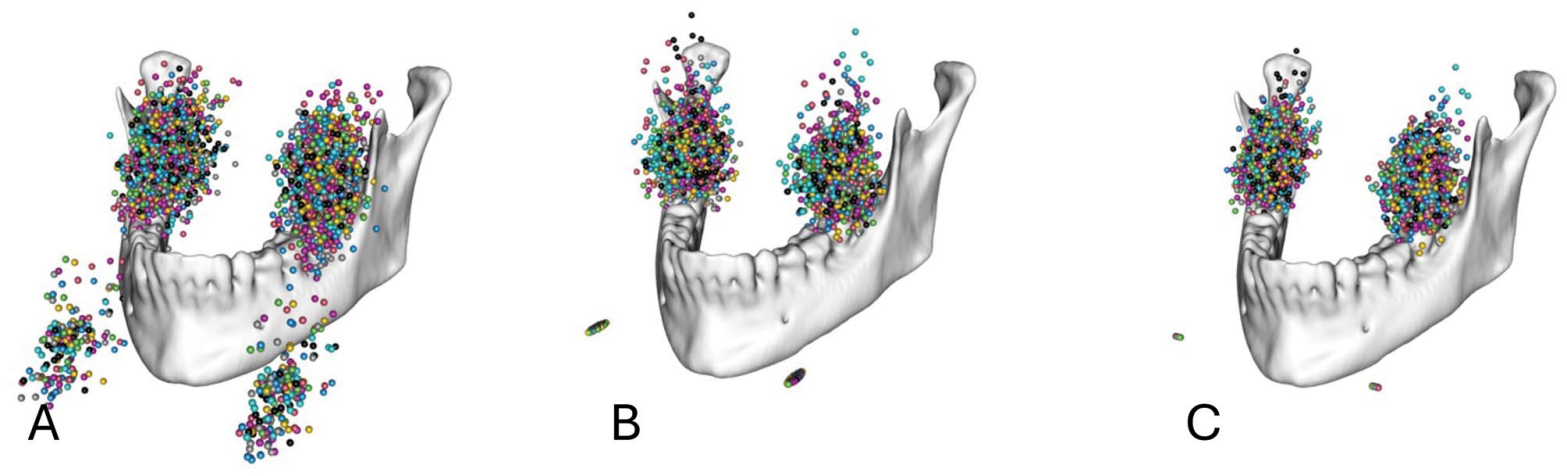
Markups for the 3 M of the maxilla in relation to the mandible (**A**) before spatial alignment, (**B**) after spatial alignment by translation to a common coordinate system, and (**C**) after spatial alignment by translation to a common coordinate system and rotation around a transversal axis defined by the chin rest

To exclude the effect of a potential suboptimal spatial positioning of the patients in the CBCT device, the landmarks were additionally rotated around an axis defined by the chin rest corners with regard to a reference individual, minimizing the least square distance between coordinates (Figs. 3 and 4).

### Determination of FOVs

Based on the distribution of the molar point clouds, cylinders were placed along the superior-inferior axis and optimized to contain 98% of all landmark coordinates.

Apart from the results of the pooled sample, the positions and dimensions of the cylinders were analyzed for sex-dependent differences. The cylinder position was determined in relation to the midpoint between the chin rest corners of the reference data set.

### Statistical analysis

All statistical tests were performed using the statistical/mathematical platform R. Statistical differences regarding sex-specific differences in height, radius, and positioning of the FOV were assessed using a permutation testing procedure. Therefore, the actual values were computed and then compared to 999 randomly redistributed group assignments. First, the actual differences regarding values describing the FOVs were computed. Then, the group affinities were scrambled and randomly assigned to each specimen, and the differences between groups were calculated. The ratio between differences in the random groups that were greater or smaller than the actual values can directly be interpreted as p-value.

## Results

A total of 1132 CBCT data sets were selected from the department’s order entry database, from patients having received CBCT imaging including the mandible as part of the clinical routine. Of these data sets, 140 met the inclusion criteria; of these 115 included the lower 3 M, 100 included the upper 3 M, and 76 data sets included both the lower and upper 3 M. The characteristics of the data sets are outlined in Table 1. A sample size analysis was not performed because of the descriptive character of the study without testing the hypothesis.

**Table 1:** Study population

	Total	Male	Female
All	140	82	58
Lower 3 M	115	66	49
Upper 3 M	100	65	35
All 3 M	76	49	27

The cylindrical volumes were determined using the 98th percentile of the markup points to account for outliers. Both cylinders, after alignment only by translation (worst-case scenario) and after optimization by both translation and rotation (best-case scenario), were calculated for all subgroups.

### Lower jaw

The diameters and heights of the determined FOVs for the inclusion of 98% of the data points of 115 lower 3 M are depicted in Table 2.

**Table 2:** Uni- and bilateral FOV dimensions in mm for the lower jaw, according to the optimization approach

		Translation only		Translation and rotation	
		Radius [mm]	Height [mm]	Radius [mm]	Height [mm]
**All**	Unilateral	36.6	35.4	35.4	23.5
	Bilateral	90.4	35.4	89.4	23.5
**Female**	Unilateral	37.6	36.4	36.0	23.4
	Bilateral	90.6	36.4	89.0	23.4
**Male**	Unilateral	36.0	34.2	34.2	23.5
	Bilateral	90.0	34.2	88.8	23.5

For the unilateral FOV, there were no significant sex-related differences regarding diameter or height. The only variable differing significantly is the position of the FOV, in both scenarios (rotation: *p* < 0.025; translation: *p* = 0.021). The resulting sex-independent FOVs were determined with dimensions (diameter × height) of 36.6 mm × 35.4 mm (translation only) and 35.4 mm × 23.5 mm (translation and rotation).

For the bilateral FOV there was a significant difference in the position of the FOV cylinders (rotation: *p* < 0.017; translation: *p* = 0.008) between the male and female sexes. The resulting sex-independent FOVs were determined to have dimensions (diameter × height) of 90.4 mm × 35.4 mm (translation only) and 89.4 mm × 23.5 mm (translation and rotation).

The sex-dependent difference in the unilateral FOV position caused a significant difference in height and radius to cover 98% of all landmarks in a bilateral FOV.

### Upper jaw

The diameters and heights of the determined FOVs for the inclusion of 98% of the data points of 100 upper 3 M were depicted in Table 3.

**Table 3:** Uni- and bilateral FOV dimensions in mm for the upper jaw, according to the optimization approach

		Translation only		Translation and rotation	
		Radius [mm]	Height [mm]	Radius [mm]	Height [mm]
**All**	Unilateral	35.6	38.6	29.2	29.9
	Bilateral	73.6	38.6	71.0	29.9
**Female**	Unilateral	35.6	37.9	30.2	29.9
	Bilateral	71.4	38.2	71.4	30.4
**Male**	Unilateral	33.0	40.4	28.8	28.4
	Bilateral	74.0	40.9	68.8	28.5

For the unilateral FOV, there were no significant sex-specific differences regarding the radius. The only value differing significantly was the position of the FOV, in both scenarios (rotation: *p* < 0.001; translation: *p* = 0.005). The resulting sex-independent FOVs were determined to have dimensions of 35.6 mm × 38.6 mm (translation only) and 29.2 mm × 29.9 mm (translation and rotation).

For both the translationally aligned and the rotationally optimized data, the positions of the bilateral FOV cylinders differed significantly (rotation: *p* < 0.001; translation: *p* = 0.003) between the sexes. The diameter differed significantly in the rotationally optimized data (rotation: *p* = 0.023; translation: *p* = 0.088).

Analogous to the lower jaw, the sex-dependent difference in the diameter of the bilateral FOV can be explained by the difference in the unilateral FOV position.

### Combined upper and lower jaw

Additionally, in 76 data sets the sizes of the FOVs for both the upper and lower M3 combined in a common FOV were analyzed. The diameters and heights of the determined FOVs for the inclusion of 98% of the data points of a combined FOV are given in Table 4.

**Table 4:** Uni- and bilateral FOV dimensions in mm for the combined upper and lower jaws, according to the optimization approach

		Translation only		Translation and rotation	
		Radius [mm]	Height [mm]	Radius [mm]	Height [mm]
**All**	Unilateral	39.8	51.7	36.8	44.8
	Bilateral	85.6	51.7	84.8	44.8
**Female**	Unilateral	42.0	51.2	34.8	45.9
	Bilateral	86.4	51.2	84.8	45.9
**Male**	Unilateral	37.0	50.2	37.8	43.8
	Bilateral	84.4	50.3	84.2	43.8

For the unilateral FOV, there were no significant differences in diameter or height depending on the male/female sex. Again, the only value differing significantly was the position of the FOV, as seen in the translationally aligned and the rotationally optimized data (rotation: *p* = 0.007; translation: *p* = 0.007). The resulting sex-independent FOVs were determined to have dimensions of 39.8 mm × 51.7 mm (translation only) and 36.8 mm × 44.8 mm (translation and rotation).

For the bilateral FOV, the only significant sex-dependent difference was seen in the position of the FOV (rotation: *p* = 0.009, translation: *p* = 0.008).

The positions of the FOVs relative to the J Morita Accuitomo chin rest as marked by the midpoint between the chin rest corners were depicted in Table 5. As a result of the data set mirroring at the midsagittal plane and the resulting symmetry of the data, the lateral position of the FOV encompassing the 3 M of both sides was always located at the midsagittal plane and equals 0. The lateral coordinates for the unilateral FOV were also only given once because of the symmetry of the data set.

**Table 5:** Positions of the FOV cylinders relative to the J Morita accuitomo 170 chin rest

		V1 (lateral)		V2 (sagittal)	V3 (axial)
		Unilateral	Bilateral		
**Lower jaw**	Female	$\:\pm\:$32.24	$\:\pm\:$0.00	–44.95	+ 13.41
	Male	$\:\pm\:$31.73	$\:\pm\:$0.00	–46.54	+ 14.65
	All	$\:\pm\:$31.96	$\:\pm\:$0.00	–45.69	+ 14.24
**Upper jaw**	Female	$\:\pm\:$23.86	$\:\pm\:$0.00	–40.97	+ 31.38
	Male	$\:\pm\:$25.67	$\:\pm\:$0.00	–43.22	+ 34.86
	All	$\:\pm\:$25.14	$\:\pm\:$0.00	–41.67	+ 33.00
**Both jaws**	Female	$\:\pm\:$28.86	$\:\pm\:$0.00	–42.46	+ 14.39
	Male	$\:\pm\:$29.37	$\:\pm\:$0.00	–44.65	+ 16.96
	All	$\:\pm\:$29.16	$\:\pm\:$0.00	–43.24	+ 16.10

## Discussion

Preoperative CBCT imaging of the third molars (3 M) after identifying potential risk factors through 2D imaging has become established as best clinical practice [13, 16]. However, this practice also results in increased radiation exposure to patients [18, 19]. To mitigate this exposure, several options are available [19], including adjustments to exposure parameters, voxel size, scan time, and, importantly, optimization of the field of view (FOV) [8, 11].

The radiation exposure associated with the FOV is influenced by both its size and its positioning relative to radiation-sensitive organs [19, 23]. Given the predominantly vertical arrangement of these organs, the height of the FOV significantly affects the effective dose [11]. In younger patients, CBCT prior to 3 M extraction is often justified when there is a risk of damaging the inferior alveolar nerve [19, 24]. Implementing a region of interest (ROI)-specific FOV could effectively reduce radiation exposure in this demographic group.

Currently, the market for CBCT devices is highly dynamic, with at least 279 devices available [21]. This variety includes different FOV options [21], but many are not based on robust clinical data. Thus, there is a pressing need for indication-specific and ROI-specific FOVs [20, 24]. To date, only two studies have examined ROI-specific FOVs in CBCT, focusing on upper impacted canines and lower third molars, respectively [21, 24].

Ilo et al. investigated an FOV specific to the lower 3 M; however, their study only assessed the FOV’s position relative to the lower second molar, rather than independently in relation to the CBCT device itself [21]. This limitation still necessitates a prior scout view to ensure proper alignment. As of now, no published data exists on patient-independent optimized FOVs for either the lower or upper 3 M.

It is important to acknowledge that our findings may be influenced by the demographics of our study population, which is predominantly Caucasian and based in southern Germany. Additionally, parameters like body height or weight were not considered, and our patient population skews young, with a median age of just 21 years. While this age group is representative of those typically undergoing CBCT scans prior to 3 M extraction, it may still distort our results [20, 21].

Our method involved marking the outlines of the tooth using only six points corresponding to the three spatial axes, combined with a cylindrically shaped FOV. This approach presents a slight risk of incomplete inclusion of teeth with atypical geometries, such as excessively curved roots or dysmorphic crowns. Furthermore, pathologies outside the determined dimensions for 3 M imaging, such as cystic lesions, may not be fully captured [24]. However, such conditions should ideally be identifiable in 2D imaging using DPT, necessitating an individualized diagnostic approach [20].

We opted not to calculate the position and dimensions of the FOV in relation to specific anatomical landmarks, aiming instead for patient-independent FOVs applicable to a broad range of patients. The chin rest of the CBCT device served as a common reference point, allowing us to overlay all data within a unified coordinate system. Potential inaccuracies arising from variations in the field of view (FoV), which was adapted to the clinical justification for each scan, were mitigated by maintaining a consistent voxel size across all scans, ensuring the feasibility and comparability of measurements within the region of interest.

The FOVs we determined represent a worst-case scenario, accounting for potential suboptimal patient positioning in the CBCT device. By employing an optimization algorithm that rotates around the chin rest, we could propose a hypothetical best-case scenario achievable through optimal patient alignment. Nonetheless, there remains a minimal risk of misalignment and undersizing in these optimized FOVs during statistical analysis.

All data were collected using the same CBCT device (J Morita Accuitomo 170), making it difficult to generalize our findings to devices from other manufacturers. However, with appropriate registration of a phantom head on different chin rests, our data could potentially be adapted for use with other devices.

To refine our analysis, we eliminated outliers by limiting our study to the 98th percentile of markups, though we did not exclude entire datasets that could have slightly distorted our results. Table 6 presents the minimal FOVs established for both worst- and best-case scenarios, along with our proposed FOVs, which include a 2-mm margin as recommended for all radiographic imaging [20].

**Table 6:** Proposed FOV dimensions for specific ROI

	Mandible unilateral	Maxilla unilateral	Mandible bilateral	Maxilla bilateral	Both jaws unilateral	Both jaws bilateral
Result [mm]	R 36.6	D 35.6	D 90.4	D 73.6	D 39.8	D 85.6
	H 35.4	H 38.6	H 35.4	H 38.6	H 51.7	H 51.7
Proposed [cm]	4 × 4	4 × 4	9.5 × 4	8 × 4	4.5 × 5.5	9 × 5.5
Idealized results [mm]	R 35.6	R 29.2	R 89.6	R 71.0	R 36.8	R 84.8
	H 23.5	H 29.9	H 23.5	H 29.9	H 44.8	H 44.8
Proposed FOV [cm]	4 × 3	3.5 × 3.5	9.5 × 3	7.5 × 3.5	4 × 5	9 × 5

For the lower 3 M, the unilateral FOV in the worst-case scenario is determined to be 4 cm × 4 cm (diameter × height), aligning well with the smallest implemented FOVs in common CBCT devices [20, 21]. The best-case scenario allows for a reduction to 4 cm × 3 cm, consistent with recommendations from Pakbaznejad Esmaeili et al., who suggest an FOV of 3.5 cm × 3.5 cm [20].

For the upper 3 M, the unilateral FOVs in the worst- and best-case scenarios are 4 cm × 4 cm and 3.5 cm × 3.5 cm, respectively, with the latter being smaller than the smallest common FOVs [20]. The bilateral imaging of both lower and upper 3 M in the worst-case scenario would require a FOV of 4 cm × 5.5 cm, while optimal patient positioning could reduce this to 4 cm × 5 cm, aligning with common small FOVs [20, 25]. However, the available FOVs on the J Morita Accuitomo 170 may be inadequate, as they are either too small (4 cm × 4 cm) or too large (6 cm × 6 cm), leading to missing anatomical structures or unnecessary radiation exposure, respectively [21].

For bilateral imaging, the worst-case FOV for the lower 3 M could be set at 9.5 cm × 4 cm, with the best case at 9 cm × 3 cm. The upper 3 M, being more medially located, allows for smaller diameters; thus, the bilateral FOVs could be set at 8 cm × 4 cm (worst case) and 7.5 cm × 3.5 cm (best case). When considering all four 3Ms, the FOVs could be adjusted to 9 cm × 5.5 cm (worst case) and 9 cm × 5 cm (best case), closely aligning with recommendations for encompassing the entire dental region. However, many CBCT devices limit FOVs to a maximum diameter of 8 cm, which may restrict the applicability of our findings regarding bilateral FOVs [5, 26].

**Fig. 5:**
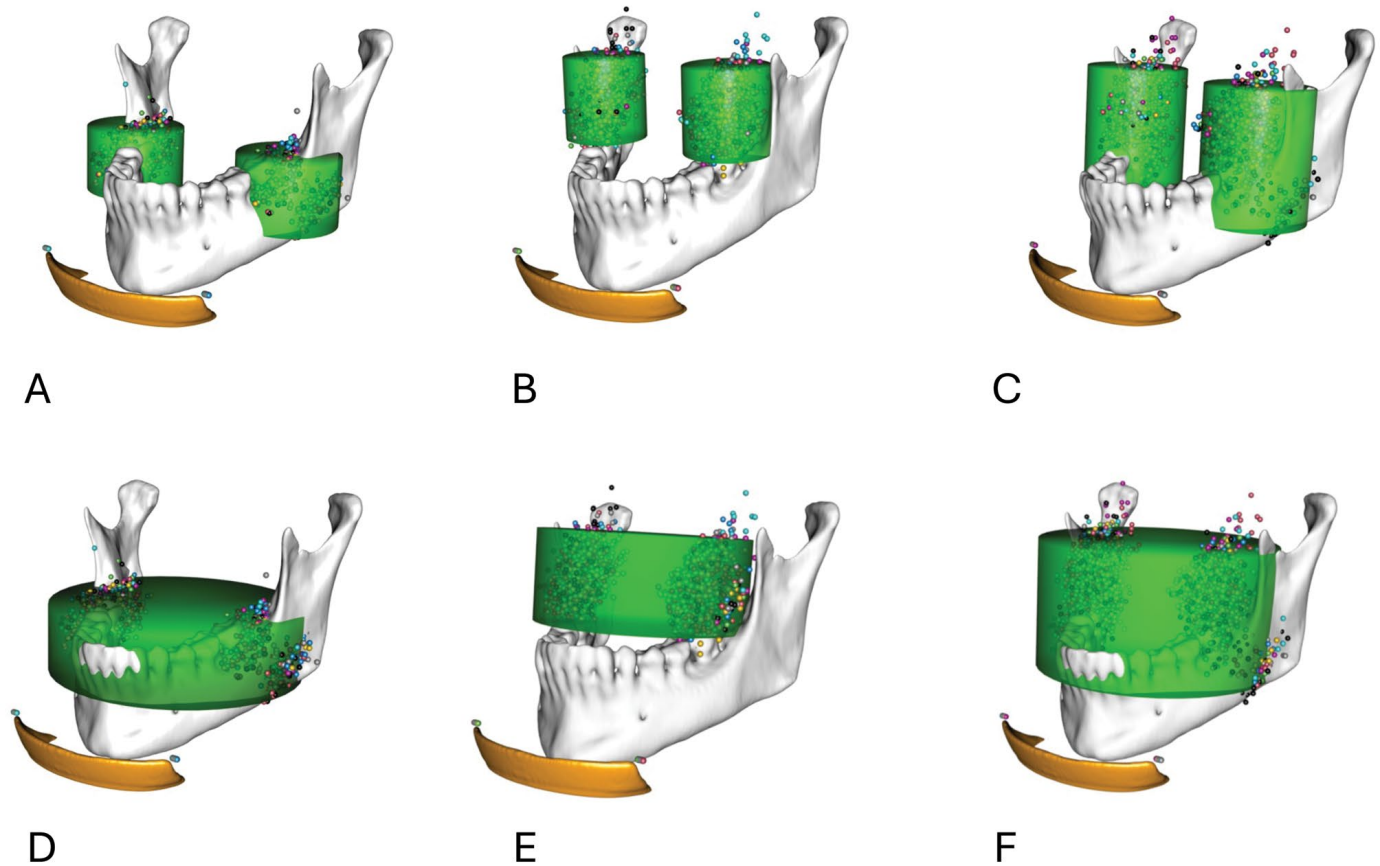
Combined FOVs for male and female data sets. **A** Isolated FoV for both lower wisdom teeth. **B** Isolated FoV for both upper wisdom teeth. **C** Isolated FoV for upper and lower wisdom teeth. **D** Combined FoV for lower wisdom teeth. **E** Combined FoV for upper wisdom teeth. **F** Combined FoV for upper and lower wisdom teeth

Our analysis also revealed significant sex-dependent differences in FOV cylinder positions, with the 3 M of male participants positioned more posteriorly and cranially. Additionally, the upper 3 M of male participants were located more laterally compared to those of female participants, a trend likely attributable to overall sex-dependent size differences. Notably, only the lower 3 M of female participants exhibited a more lateral localization (Fig. 5). To our knowledge, there is currently no conclusive data on sex-dependent localization of 3Ms [5, 27].

## Conclusion

By implementing the results of the performed study, the volume for a single 3 M image was defined as sex-dependent, allowing for more accurate localization and assessment of wisdom teeth based on gender differences. Further research should aim to assess the adaptability of the findings to various commercially accessible CBCT devices and diverse demographic populations.

## Data Availability

No datasets were generated or analysed during the current study.

## Abbreviations

CBCT: Cone-Beam Computed Tomography
CT: Computed Tomography
DJD: Degenerative Joint Disease
ED: Effective Dose
FOV: Field of View
ROI: Region of Interest
TMJ: Temporomandibular Joint
